# Resource Use in Small Island States

**DOI:** 10.1111/jiec.12100

**Published:** 2014-02-12

**Authors:** Fridolin Krausmann, Regina Richter, Nina Eisenmenger

**Keywords:** industrial ecology, material flow analysis (MFA), production and consumption, resource management, societal metabolism, trade and environment

## Abstract

Iceland and Trinidad and Tobago are small open, high-income island economies with very specific resource-use patterns. This article presents a material flow analysis (MFA) for the two countries covering a time period of nearly five decades. Both countries have a narrow domestic resource base, their economy being largely based on the exploitation of one or two key resources for export production. In the case of Trinidad and Tobago, the physical economy is dominated by oil and natural gas extraction and petrochemical industries, whereas Iceland's economy for centuries has been based on fisheries. More recently, abundant hydropower and geothermal heat were the basis for the establishment of large export-oriented metal processing industries, which fully depend on imported raw materials and make use of domestic renewable electricity. Both countries are highly dependent on these natural resources and vulnerable to overexploitation and price developments. We show how the export-oriented industries lead to high and growing levels of per capita material and energy use and carbon dioxide emissions resulting from large amounts of processing wastes and energy consumption in production processes. The example of small open economies with an industrial production system focused on few, but abundant, key resources and of comparatively low complexity provides interesting insights of how resource endowment paired with availability or absence of infrastructure and specific institutional arrangements drives domestic resource-use patterns. This also contributes to a better understanding and interpretation of MFA indicators, such as domestic material consumption.

## Introduction

Material flow accounts provide aggregate information on the physical characteristics of national economies. In recent years, a growing number of national case studies have studied the development of material flow indicators over longer periods of time, investigating the emergence of industrial metabolic profiles (e.g., Gonzalez-Martinez and Schandl 2008; Singh et al. 2012; Wang et al. 2012; Schandl and West 2012). The size and composition of material-use patterns of a national economy is influenced by a broad range of biogeographic and socioeconomic factors, from resource endowment to industrial structure and wealth (Weisz et al. 2006). In larger economies, the interplay of different factors in shaping the metabolic profile is often obscured by the complexity of the physical economy and it is difficult to identify the different factors that lead to an observed material flow pattern; a discussion of trends in resource use often remains limited on macro trends, such as growth in population and gross domestic product (GDP). In this article, we have chosen two small island economies with a comparatively simple structure of industrial production, trade, and resource-use patterns as case studies to gain a better understanding of how national material-use patterns emerge.

As Deschenes and Chertow (2004) have pointed out, island systems are an interesting and manageable object for industrial ecology research. Islands are, in many respects, closed and bounded systems, often with few locally occurring and limited resources. At the same time, from a material flow analysis (MFA) perspective, they often appear as open economies, which depend on the exploitation of a few key resources for export production and on imports for a large part of their domestic needs and final consumption. This makes them highly vulnerable to both global economic change and domestic environmental degradation; in the island context, sustainability challenges are generally much more immediate than at a continental scale, as the example of Nauru and its rise and fall resulting from the exploitation of its phosphate deposits illustrate (Gowdy and McDaniel 1999).

Industrial ecologists have used islands as case studies mostly with a focus on waste flows (Saito 2013) and industrial symbiosis (Ashton 2009; Eckelman and Chertow 2009). This article applies economy-wide material flow accounting to investigate the long-term development of material extraction, trade, and consumption in two small island states, Iceland and the Republic of Trinidad and Tobago (short: Trinidad and Tobago or TT), in the period from 1961–1962 to 2008. On a global scale, the two small countries seem to be of little significance, with a population of only 0.36 and 1.3 million (mio), respectively; together, they only represent 0.02% of the global population and contributed 0.07% to global GDP in 2008. But, the two high-income economies (table1) stand out in terms of their resource use and share a number of unique metabolic characteristics. Quite untypical for small Island states, both countries are endowed with abundant energy resources, although of different types: Whereas Iceland is rich in renewable hydropower and geothermal heat, TT exploits petroleum and natural gas deposits. Today, the two countries stand out in terms of per capita resource use and emissions: Both have an exceptionally high level of energy use and energy intensity of the economy (table1). In 2005, TT and Iceland were (after Kuwait) the two countries with the highest per capita primary energy consumption worldwide and TT was also one of the top five emitters of carbon dioxide (CO_2_), measured in terms of emissions per capita (table1). Together, both countries contributed 0.16% to global CO_2_ emissions in 2008. These unique resource-use patterns, and being small economies with a narrow, but economically important, resource base and a high significance of trade for domestic supply and economic development, make Iceland and TT interesting cases to study resource-use patterns in relation to resource endowment, industrial production, and trade. These structural similarities, paired with quite different specific patterns of resource endowment, biogeographic setting, and economic history, make them also interesting objects for comparative discussion.

**Table 1:** Socioeconomic characteristics of Iceland and Trinidad and Tobago in global comparison

*Indicators*	*Iceland*	*Trinidad and Tobago*	*EU27*	*Latin America and Caribbean*	*World*
Population density (persons/km^2^)	3	259	119	29	52
Population growth (annual %)	1.9	0.4	0.4	1	1.2
GDP per capita, PPP (in US$ of 2005)	36,656	24,151	28,486	9,962	9,630
GDP growth (annual %)	1.3	2.7	0.3	4.1	1.3
Energy use (tonnes oil equivalent per capita)	16.5	14.5	3.5	1.3	1.8
Energy use (kilogram oil equivalent per $1,000) GDP (PPP, in US$ of 2005)	451	601	123	133	183
Electric power consumption (kilowatt-hours per capita)	50,067	5,646	6,384	1,910	2,858
CO_2_ emissions (metric tonnes per capita)	7	37	8	3	5
CO_2_ emissions (kilogram per $ GDP [US$ of 2000])	0.19	3.41	0.39	0.59	0.79
Imports of goods and services (% of GDP)	47	37	40	26	30
Exports of goods and services (% of GDP)	44	64	41	25	30

Data refer to 2008. *Source*: the World Bank Group (2012).

km^2^ = square kilometer; GDP = gross domestic product; PPP = purchasing power parity; CO_2_ = carbon dioxide; EU = European Union.

The article investigates the development of material and energy flow patterns in the two countries. It applies MFA methods to analyze changes in direct material use during a period of almost five decades from 1961 to 2008. We quantify extraction, trade, and material consumption of biomass, fossil energy carriers, ores, and nonmetallic minerals and calculate aggregate MFA indicators. The relatively simple structure of the economy of the two countries, which is dominated by a few resource-intensive, export-oriented industrial sectors, allows for discussing different factors influencing the emergence of their above-average level and specific patterns of material and energy use. We will discuss how the current metabolic profile emerged and how specific biogeographic and socioeconomic factors drove material flow development. We explore how the two island states make use of their narrow resource base and how they manage their resources. Specific attention will be given to the role that resource endowment, infrastructure, trade, and institutional settings played for the development of material flows. The example of the two small open economies also yields interesting insights concerning the interpretation of material flow indicators in relation to production and consumption patterns (Weisz and Steinberger 2010).

The next section provides a brief, but concise, presentation of the applied MFA methods and data sources. This is followed by the presentation of key results and a discussion of material-use patterns, first for TT and then for Iceland. In this section, we give a detailed assessment of the development of material flows with a special focus of the key resources and their extraction, processing, and trade. The third section compares the metabolic profile of the two countries, puts them in a global context, and discusses the interpretation of material-use indicators with respect to sustainable development. We conclude with some general comments on the meaning of material-use indicators and the role of resource endowment and trade for the development of small island states.

## Methods

The MFA database for the two countries covers the period from 1961–1962 to 2008.^1^ It includes data on direct material flows for domestic extraction (DE), imports and exports, the physical trade balance (PTB; imports minus exports), and domestic material consumption (DMC; DE plus PTB, also termed material use in the text) and distinguishes between 65 material groups, which are aggregated to five main material groups: biomass; fossil energy carriers; metals; nonmetallic minerals; and nonspecified materials (only for trade). The presented material flow accounts are based on the methodological principles and compilation guidelines developed by Eurostat (2009). They use national and international statistical data sources and standard estimation procedures for flows not covered in statistics (Krausmann et al. 2009; Fischer-Kowalski et al. 2011). We used population data from the Food and Agriculture Organization (FAO) Statistics Division (FAOSTAT) (2010) to calculate per capita values and GDP data from the World Bank Group (in constant 2005 USD) to calculate material intensity (DMC per unit of GDP).

### Domestic Extraction

Extraction of biomass was calculated on the basis of the FAO agricultural and forestry statistics (FAOSTAT 2010). Additionally, we used data from the Statistical Office of Iceland to fill gaps for the DE of fish, hunted animals, and forage. Harvested crop residues and biomass grazed by livestock were estimated following standard procedures and region- and time-specific coefficients (Krausmann et al. 2008). DE of fossil energy carriers was compiled from the International Energy Agency (IEA) and United Nations (UN) energy statistics (IEA 2010; UN Statistics Division [UNSD] 2011) for TT. Iceland did not exploit any oil or gas deposit in the observed period. Data on the extraction of ores and nonmetallic minerals were derived from U.S. Geological Survey (USGS) minerals yearbooks (e.g., USGS 2008) and the UN's industrial commodities statistics (UN 2010). Both countries did not exploit ores during the observed period. The DE of nonmetallic minerals used for construction was insufficiently represented in national and international statistical sources. We estimated the extraction and use of sand, gravel, crushed stone for the production of cement, and production of concrete and asphalt based on information on the production and consumption of cement (e.g., Cembureau 1998) and bitumen (IEA 2010) as well as appropriate coefficients provided in previous studies (Steinberger and Krausmann 2011; Krausmann et al. 2009; Eurostat 2009).

**Figure 1:**
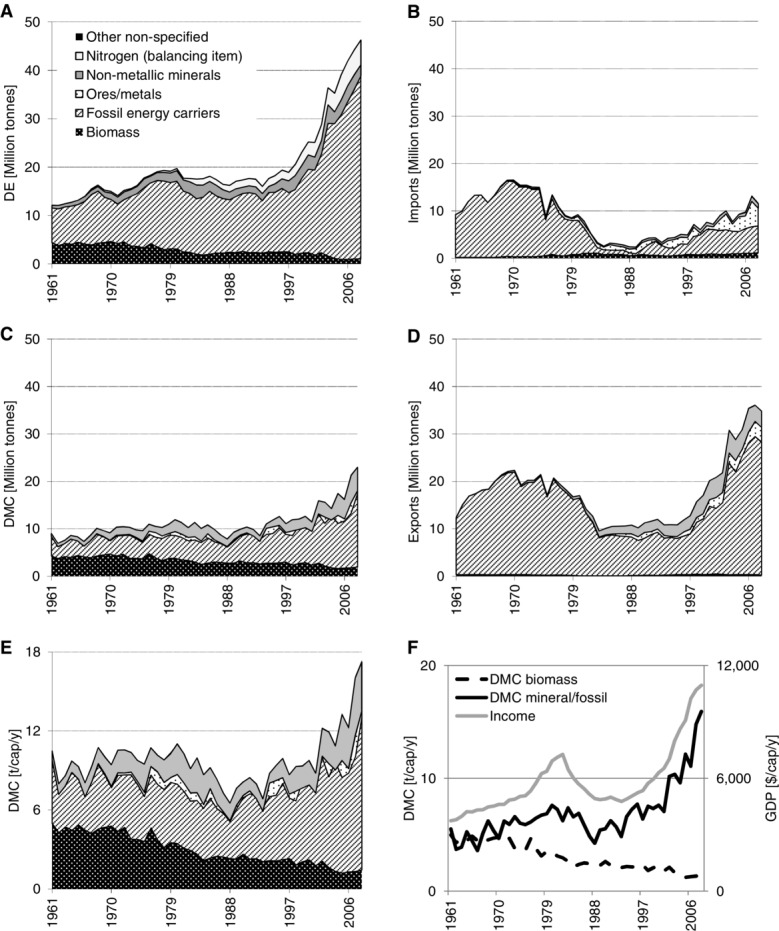
Material flows in Trinidad and Tobago by main material groups: domestic extraction (DE), imports, exports, and domestic material consumption (DMC) in million tonnes per year (10^6^ t); DMC in tonnes per capita per year (t/cap/yr); and income (gross domestic product in constant dollars of 2005 per year [$/cap/yr]) and DMC of biomass and of mineral/fossil materials in t/cap/yr.

TT produces large amounts of nitrogen fertilizer (ammonia) for export. Raw material for this production is atmospheric nitrogen, which is (as with the extraction of other atmospheric gases and water) not accounted for as DE in material flow accounts, but is considered a so-called “balancing item” (Eurostat 2009).^2^ We have quantified the extracted nitrogen on the basis of stoichiometric composition of the produced compounds and included it as a separate item in DE (figure 1A).

### Trade

We used the FAO data for trade with products from agriculture and forestry (FAOSTAT 2010) and IEA (2010) data for trade with fossil energy carriers and derived products. Trade with ores, metals, nonmetallic minerals, and products thereof was compiled from the UN's Comtrade database (UNSD 2012). For Iceland, we cross-checked international trade data with data available from Statistics Iceland (2011).

## Material Flows in Trinidad and Tobago

TT is an archipelagic state in the Caribbean. It consists of two main islands, Trinidad and Tobago. Trinidad comprises approximately 94% of the total area and is the home of 96% of the population and all industries. On a total area of only 5,100 square kilometers (km^2^), TT was inhabited by 1.3 mio people in 2008. Its population density is high and grew from 167 capita per square kilometer (cap/km^2^) in 1961 to 266 cap/km^2^. It is one of the wealthiest states in the Latin American and Caribbean region, with per capita GDP measured in purchasing power parity (PPP) amounting to US$24,000 in 2008 (table1).

## Results

Figure1A shows that total material extraction (DE) grew from 12 to 46 mio tonnes (Mt). DE is dominated by the large and growing fraction of fossil energy carriers and a rising amount of atmospheric nitrogen (see “*Methods*” section). Biomass, still an important domestic flow in the 1960s, loses significance. The share of fossil energy carriers in DE, first petroleum and later mostly natural gas, rose from 59% to 82%.

TT has large imports (figure 1B), and also these are dominated by fossil energy carriers, although their share is declining from 98% in the 1960s to 40% to 50% in the 2000s. Next to fossil energy, TT is importing considerable amounts of metals, mostly iron ores for an iron and steel industry, which was established to diversify the oil base economy (Holton 1994). Although biomass imports are dwarfed, compared to fossil and metal materials, it is important to note that TT depends, to a growing degree, on biomass imports for its supply of food. Per capita food imports increased from 0.2 to 0.7 tonnes (t) per year (t/yr) in 2008 and reached a size similar to biomass extraction (0.8 t per capita per year [t/cap/yr]). Exports (figure 1D) follow a similar trend as imports, but are significantly larger. Exports tripled in the period and are dominated by fossil energy carriers (98% to 80%). Next to fossil fuels (FFs), TT exports considerable amounts of mineral materials, mostly fertilizers (10% in 2008) and iron and steel (9% in 2008). Per capita export volume almost doubled, from 14 to 27 t/cap/yr, and even exceeded DE until the late 1970s. TT has been a net exporter of materials during the whole period. The terms of trade (average price of traded goods) of its exports was, however, comparatively low, and an initial monetary trade surplus dwindled in the 1970s. With falling oil prices in the 1980s, the monetary trade balance even became negative for almost a decade. Only with the exports of gas and related products and high energy prices in the last two decades did TT again begin to achieve a high monetary surplus from its foreign trade (see figure S1 in the supporting information available on the Journal's website).

DMC has been increasing (from 9 to 23 Mt/yr) and was initially dominated by biomass and fossil materials and, in later periods, also by nonmetallic minerals (figure 1C). DMC of biomass was declining, whereas the DMC of mineral and fossil materials closely followed the development of GDP (figure 1F). TT has a comparatively high per capita DMC, which declined during the long recession of the 1980s, but multiplied during the subsequent period of high economic growth. Between 1993 and 2008, TT's DMC increased from 7 to 17 t/cap/yr, a level far above global average material use (9 t/cap/yr) and even higher than, for example, the average of the European Union (EU)27 (12 t/cap/yr). In particular, the per capita DMC of fossil energy carriers is high, with 12 t/cap/yr. As a result, TT also has very high per capita CO_2_ emissions from FF combustion and cement production (37 t/cap/yr).

## Discussion

In the case of TT, we observe the transition from a biomass- to an oil-based economy. The Caribbean island state was a British colony until 1958. Until World War II, the backbone of the economy was the production of sugar and, to some degree, also cacao and coffee for export (Meditz and Hanratty 1987), and biomass dominated material extraction and use. At the beginning of the observed period, in 1961, biomass still accounted for over one third of all domestic extraction. The second important resource was petroleum, which has been exploited since the late-nineteenth century (Furlonge and Kaiser 2010). Already in the 1930s, petroleum extraction exceeded 1 Mt, increased until 1961 to 6 Mt, and doubled again to 12 Mt in 1977, when TT reached its peak production; since then, petroleum extraction declined by 35%. But, domestic petroleum resources were only one factor driving the growth of petroleum industries in TT. The Mandatory Oil Import Quota Program of the United States, established in 1959, limited oil imports from certain countries to the United States, in particular, from the Near East to limit U.S. dependency on oil imports and protect the domestic oil industry. TT was exempt from these restrictions, had a deep-sea harbor, and a favorable geographic location in the Caribbean in proximity to the United States (Holton 1994). Additionally, after gaining independence, TT followed a policy of “industrialization by invitation,” which based industrial development on investments by multinational companies (Lewis 1950). In 1956, Texaco was the first investor to take advantage; others, such as Shell, followed. Texaco bought the national Trinidad oil company and began to ship oil from its fields in the Near East and process it in the TT refinery facilities to residual fuel oil, which was then exported to the United States as feedstock for the petrochemical industry. This triggered TT's emergence as an important refinery location and one of the world's largest trans-shipment centers (Holton 1994). This development is reflected in the MFA data: Already in 1961, petroleum imports exceeded domestic extraction and they increased to 15 Mt in 1970, twice the amount of domestically extracted petroleum. The economic benefits of this development were, however, limited and the trade surplus dwindling. The Black Power uprising in 1970 changed the political landscape in TT and, as a consequence, also brought a change in foreign investment politics and the nationalization of important industries (Tewarie and Hosein 2003). Oil imports rapidly declined to almost zero in the early 1980s, and, increasingly, the domestic resource base was exploited. Though TT's economy benefitted from the high oil prices of the 1970s (Auty and Gelb 1986), falling oil prices, paired with the depletion of domestic stocks and a lack of new discoveries, hit TT's economy and the country fell into a fierce recession, with income falling by one third between 1983 and 1989 (figure 1F) (Byron 2007). It took until 2003 for the income level of the years before the crises to be reached again; since then the economy has been growing at high rates, mainly based on large revenues from natural gas extraction (figure 1F).

For a long time, natural gas was more of a waste product of petroleum extraction than a valuable resource, because TT lacked the infrastructure to use large quantities domestically or to export gas. This changed in the late 1970s, when the fertilizer industry and other natural-gas–using industries expanded. Natural gas extraction was further boosted by the opening of the first plant for the production of natural gas liquids in 1999, which allowed for direct exports of large amounts of gas (Furlonge and Kaiser 2010). In the 10 years between 1998 and 2008, natural gas extraction multiplied by a factor 4.5. In 1999, natural gas extraction began to exceed petroleum extraction, and in 2008, roughly 30 Mt were extracted. Currently, TT is the fifth-largest exporter of natural gas liquids (NGLs), with two thirds being shipped to the United States (USGS 2008). The second important natural-gas–based industry was the production of nitrogen compounds for export, which increased quickly from 0.5 Mt in 1980 to 5 Mt in 2008 (for comparison: petroleum extraction was 8 Mt in 2008). In 1985, TT became the world's second-largest exporter of nitrogen fertilizer after the USSR (Meditz et al., 1987). Another element of TT's strategy of industrial diversification in the 1970s and 1980s was the establishment of an iron and steel industry. This industry, which exclusively operates on imported ores from Brazil, is reflected in the growing imports and exports of ores and metals, respectively (UNSD 2012). Iron ore imports reached 5 Mt in 2007 or 37% of total imports. Roughly two thirds of the imported iron ores and concentrates are exported as reduced iron or steel billets, and the remainder stays as waste in TT.

With the growing significance of the petroleum industry, rising labor costs, and falling sugar prices, sugar production and export rapidly declined (Prorok 2010). Until the early 1970s, approximately 2.5 Mt of sugar cane were harvested in TT, with sugar cane and related residues accounting for 86% of biomass DE; with the decline of the sugar industry, sugar cane production fell to approximately 1 Mt in 1985 and then again to the current level of 0.4 Mt after 2002. Quite surprisingly, in this last period, TT even became a net importer of sugar. The reduction of sugar production is the reason for the overall trend of a declining DE of biomass; TT eventually became a net importer of biomass in the 1970s and imports the largest share of its staple foods and wood. Net per capita imports of biomass increased to 0.7 t and exceeded domestic production of biomass. Biomass consumption per capita declined from 4 to 5 t/cap/yr in the 1960s to less than 0.5 t in the twenty-first century. The high per capita biomass DMC in the 1960s was a result of export production: Of the harvested sugar cane, only a small fraction of 5% to 10% is actually exported in the form of refined sugar; all the residues remain within the producing country, where they are partly used or burned and add to DMC.

TT has a very high and still rising level of DMC of 17 t/cap/yr in 2008, far above the Latin American average (table2). The high level of material use, however, does not reflect a particularly material-intensive lifestyle or high household consumption^3^; it is rather a result of TT's production structure: Its economy is based on material- and energy-intensive industries, which are based on domestic energy resources and imported raw materials, but produce only for export. This general pattern of an overly proportionally high DMC characterized TT's metabolic profile from the colonial period, when sugar exports resulted in very high per capita DMC of biomass, to the present, when exports of steel, fertilizer, petroleum products, and NGL boost per capita DMC. In the case of TT, the export industry contributed to rapid economic development. TT is generating a large share of its GDP with these export-oriented industries and its per capita income is one of the highest in Latin America, with low unemployment and poverty rates. But, the resource-intensive export industries also have negative side effects. They make the economy highly vulnerable, as the decline in per capita GDP resulting from falling oil prices in the 1980s indicates (Auty 1986) (figure 1E) and cause considerable ecological costs: TT has one of the highest levels of per capita CO_2_ emissions worldwide, and oil and gas extraction as well as heavy industries create large, often hazardous waste flows and impose a high risk of accidents and oil spills.

**Table 2:** Material flows of Iceland and Trinidad and Tobago in international comparison

*Indicators*	*Iceland*	*Trinidad and Tobago*	*EU27*	*Latin America*
DE (t/cap/yr)	14.0	34.7	10.0	13.7
Import (t/cap/yr)	15.1	8.8	6.3	1.0
Export (t/cap/yr)	6.1	26.2	3.9	2.0
DMC (t/cap/yr)	23.0	17.4	12.4	12.6
Material intensity (kg/$)	0.60	1.59	0.67	2.54

Data for Iceland and Trinidad and Tobago refer to 2008; averages for the European Union (EU27) and the Latin America region for 2010 are from the GLOMETRA global MFA data set (Schaffartzik et al. 2014), and import and export include intraregional trade (total trade volume by total population). Material intensity was calculated using gross domestic product in US$ of 2000 from the World Bank Group (2012).

DE = domestic extraction; DMC = domestic material consumption; t/cap/yr = tonnes per capita per year; kg/$ = kilograms per dollar.

## Material Flows in Iceland

### Results

Iceland is a Nordic European island country in the North Atlantic; with an area of 103,000 km^2^, it is much larger than TT, but has a much smaller population (315,000 in 2008). Population density is extremely low, but increased from 1.7 to 3.0 cap/km^2^ between 1961 and 2008. Because of the cold climate, Iceland is barely suitable for agriculture. Iceland's vast inland glaciers are the origin of large glacial rivers, and Iceland is geologically highly active. As a consequence, Iceland is highly endowed with hydropower and geothermal heat (Central Bank of Iceland 2008). Iceland has developed from one of the poorest countries in Europe to one of the highest-income countries. It had a per capita GDP of US$33,200 PPP in 2008 (table1).

**Figure 2:**
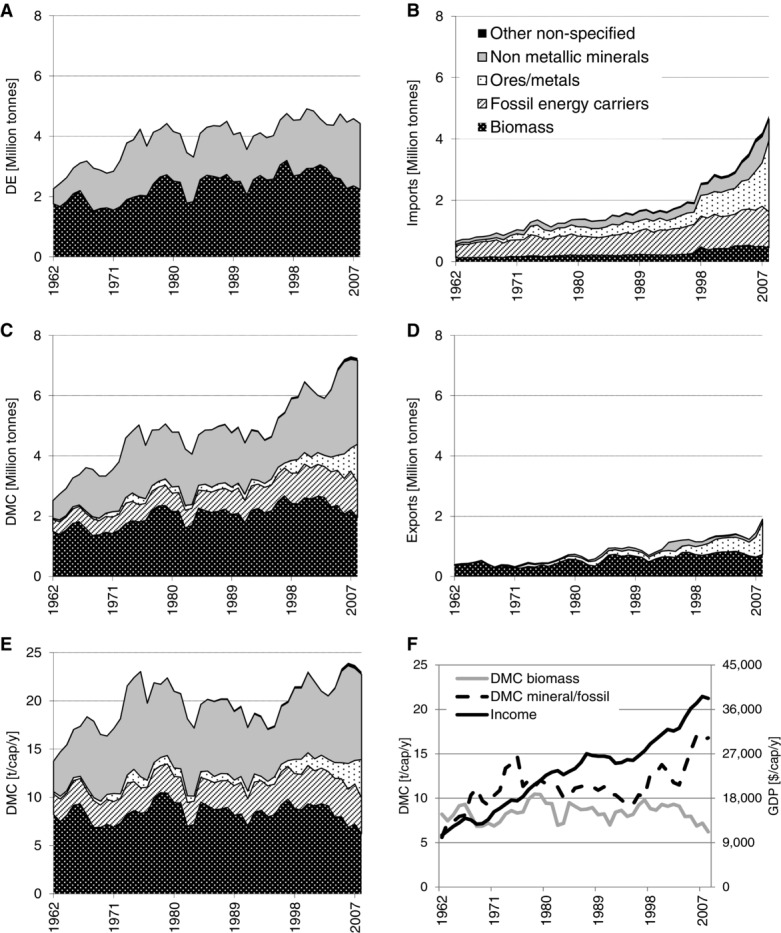
Material flows in Iceland by main material groups: domestic extraction (DE), imports, exports, and domestic material consumption (DMC) in million tonnes per year (10^6^ t); DMC in tonnes per capita and year (t/cap/yr); and income (gross domestic product in constant dollars of 2005 per year [$/cap/yr]) and DMC of biomass and of mineral/fossil materials in t/cap/yr.

Figure2A shows that Iceland's domestic material extraction is dominated by biomass and nonmetallic minerals for construction. Ores and fossil energy carriers are not extracted at all on the island. Biomass extraction doubled from 1.6 to 3.2 Mt in 1997 and has since declined. The increase in extraction was solely the result of rising fish catch, the share of which, in total DE, increased from 40% to 70% in 1997, but has since declined. In 2008, fish catch still made up 58% of biomass DE. Most of the remainder is biomass grazed by livestock; crop and vegetable production is of very minor significance because of adverse climatic conditions. Iceland is importing large amounts of biomass, and, as in TT, food supply is largely based on imported crops and livestock products. Per capita imports of biomass grew from 0.7 to 1.8 t/cap/yr, whereas exports of biomass, almost exclusively fish, ranged between 1.3 and 3 t/cap/yr. DE of nonmetallic minerals, mostly sand, gravel, and stones for construction, surged between 1961 and 1975 from 0.7 to 2.2 Mt; from then on, it fluctuated between 1.5 and 2.2 Mt/yr.

Imports and exports grew at a fast pace, following a similar trend as GDP. Imports increased from 0.4 to 4.8 Mt (figure 2B) and began to exceed domestic extraction in 2008. Exports increased from 0.3 to 1.9 Mt (figure 2D). This makes Iceland a net importer of materials; net imports increased from 0.1 to 2.8 Mt. Iceland depends, to a very high degree, on imports for many products and its supply of key resources. The import dependency of food has already been mentioned above, but also large amounts of fossil energy carriers, metals, and consumer products are imported. Iceland imports all its FFs, almost exclusively petroleum for the large fishing fleet and motorized land transport. FF imports are large and increased from 2.2 to 4.3 t/cap/yr. The high imports of metal materials not only include products such as cars or ships, but the largest fractions are bauxite (35% of total imports) for alumina and iron for ferroalloy production. Exports increased from 1.8 to 6.0 t/cap/yr in 2008 and consisted mostly of fish (36%), aluminum and ferrosilicon (55%), and some specific nonmetallic minerals (sand and diatomite). Though Iceland has been a considerable net importer of materials during the observed period, its monetary trade balance was more or less balanced until the late 1990s, thanks to very favorable terms of trade, which are much higher for exports than for imports (figure S1 in the supporting information on the Web).

Iceland's DMC has more than tripled to 7.2 Mt (figure 2C). This growth occurred mainly during two periods: between 1961 and 1974 and between 1995 and 2007. In the 20 years between 1974 and 1995, DMC remained fairly constant. Overall, growth led to doubling of material use per capita from 12 to 23 t/cap/yr (figure 2E). In international comparison, Iceland has reached a very high level of per capita material use, with the average in the EU (EU27) being only 14 t/cap/yr (table2).

### Discussion

As in Trinidad and Tobago, the economy of Iceland is based on the exploitation of a few key resources. Except for its abundant hydropower and geothermal heat, Iceland lacks natural resources—which is reflected in its pattern of material extraction: Only three materials (sand and gravel, grazed biomass, and fish) account for 95% of domestic extraction; historically, the Icelandic economy depended heavily on fishing, which still provides 40% of export earnings and employs 5% to 7% of the workforce (Statistics Iceland 2011). Fishing is still a main industry, but its importance has diminished from an export share of 90% in the 1960s to 40% in 2006. The economy diversified, and next to fish, the most important export products are currently aluminum and ferrosilicon.

In the observed period, Iceland experienced high economic growth and income increased from US$9,900 in 1961 to US$38,200 in 2008. Iceland developed from one of the poorest countries in Europe to one of the richest economies in the world. After a period of economic growth from 1961 to 1987, which was driven by the expansion of the fishing industry and rising world market prices for fish, Iceland's successful economic development was interrupted by several periods of recession from 1966 to 1969 and from 1982 to 1983 (Agnarsson 2000). In the years 1988–1993, Iceland was hit by a major economic crisis and income declined from US$27,000 to US$25,000; in 1994, the economy began to grow again at high rates until 2008. Iceland was one of the industrial countries hit hardest by the 2008 economic crisis; the economy went into strong recession for two consecutive years and GDP declined by 10% between 2008 and 2010, but Iceland recovered quite well, and in 2011, still was the 25th richest country in the world.

Iceland's economy was traditionally based on the exploitation of marine fish stocks and the export of raw and processed fish (Central Bank of Iceland 2010). Fish catch increased from 0.6 Mt in 1961 to 2.2 Mt in 1997 and has since declined as a result of decreasing stocks. In 2008, total fish catch was down to 1.2 Mt. Iceland has long been among the top ten fish-exporting countries (measured in mass flows). Export per capita fluctuated between 1.3 and 3.0 t/cap, and domestic final consumption of fish increased from 60 to 90 kg/cap/yr (FAO 2010), ten times above the global average. Measured in mass units, only between 35% and 50% of the total fish catch are exported. The large difference between total fish catch and exports is, however, not a result of the high level of household consumption of fish, but a result of processing. More than half of the total catch undergoes a treatment called “reduction,” which produces fishmeal with a very low moisture content and thus a much lower mass as the fresh catch.^4^

The observed increase in fish production was based on the expansion of fishing grounds and a cause for international conflict (the so-called “Cod Wars” with Great Britain in 1961, 1973, and 1976) (Jóhannesson 2004). Fish production and exports are highly vulnerable to declining fish stocks, as the large fluctuations in fish capture indicate. The massive slumps in fish production (and total biomass DE; figure 1) in 1965, 1981, and 1988 were a result of overfishing. In 1965, the population of Atlantic herring collapsed and catch shifted toward capelin and cod. But, also, these populations suffered from overfishing as a result of the industrialization of fish catch, which resulted in the catch slumps of 1981 and 1988 (Matthíasson 2003). Through the high significance of fishing for Iceland's economy, the various fishing crises contributed to economic recession. Iceland approached these problems by introducing a quota system for fish catch (“Individual Transferable Quota”) (Arnason 1993), based on principles derived from population ecology, which has improved the situation (Agnarsson and Arnason 2003). The fishing industry is also responsible for a considerable share of Iceland's large FF imports (23%) for providing the large fleet with fuel (Statistics Iceland 2012).

Next to fish, Iceland is endowed with abundant hydropower and geothermal heat. The use of these energy sources has a long tradition in Iceland. Iceland is covering all of its domestic electricity demand and over 90% of heat for households from these renewable resources. But, the use of hydropower and heat was, until the 1970s, restricted to a limited domestic demand (Orkustofnun 2006). Electricity cannot be exported because of a lack of a submarine cable to the European mainland, which is a technical challenge and requires large investments (Landsvirkjun 2012). Instead, Iceland pursued another pathway of exporting its energy resources: An industry with a high electricity demand is the aluminum industry. In order to boost foreign investment, the Icelandic government invited aluminum multinationals by offering guarantees for power supply, low power rates, and good conditions with respect to taxation and industry location (Skúlason and Hayter 1998). In 1969, the first aluminum smelter was put into operation by Alcoa (USGS 1970). Iceland began to export electricity in the form of aluminum and ferrosilicon. Because Iceland does not have any iron or bauxite deposits, all raw materials are imported from the United States, Australia, and Ireland (UNSD 2012). As a consequence, bauxite imports rose from 0.4 to 1.6 Mt with sharp increases in 1998 and 2007, when new plants increased the production capacity (USGS 1999, 2008). Iron imports are much smaller, but also amounted to 150,000 t in 2008. Bauxite and iron, for the production of ferrosilicon, accounted for 47% to 76% of all metal imports. The mass of the export products is much smaller than the imported raw materials (an average of 60% of the imported mass is exported), and large amounts of slag and by-products contribute to a comparatively high DMC of metal products in Iceland (4 t/cap in 2008, of which approximately 2.7 t were production wastes). Electricity is sold at an extremely low price to industry, and exploitation is increasing fast. Hydropower and geothermal heat are clean and renewable resources; however, their potential is also limited. Electric power potential from hydropower and geothermal sources is currently estimated at 50,000 gigawatt-hours per year, taking into account feasibility and environmental considerations. Currently, only 20% of these resources are exploited (Orkustofnun 2006). Plans for further expansion, among others by constructing an underwater cable to the UK, exist and are subject to controversial discussion because new dams, power stations, and industrial facilities are expected to have considerable impacts on Iceland's ecosystems and remaining wilderness areas (see Kristmannsdóttir and Ármannsson 2003; Giudice 2008; Valfells et al. 2004). Even though Iceland's physical economy is largely based on the exploitation of renewable resources, the heavy draw of the export economy on these resources has its environmental toll and causes emissions and problematic production wastes, has led to overexploitation and unsustainable management of ecosystem services, and conflicts with nature conservation goals. In spite of the unrivaled reliance on renewable energy for its domestic supply, the per capita CO_2_ emissions of Iceland are only slightly below the European and far above the global average (table 1) (Environment Agency of Iceland [EAI] 2011).

## Comparative Discussion and Conclusions

Both TT and Iceland are small open economies with a narrow resource base, a comparatively simple structure of their economy, and a very specific metabolic profile. Their high domestic extraction is dominated by only a few major materials: natural gas, petroleum, and nitrogen in TT and fish and nonmetallic minerals for construction in Iceland. Abundant energy resources, in both cases, have triggered a shift from biomass-based industries toward the establishment of large energy- and material-intensive petrochemical and metal-processing industries. Most of the extraction, but also a considerable part of the imported materials, is used in export production, and per capita exports are very high, at 26 t/cap/yr in TT and 6 t/cap/yr in Iceland. Nevertheless, both countries also have an exceptionally high level of domestic material and energy use,^5^ whereas they are both, to a large degree, dependent on imports of food and consumer goods for their domestic final consumption. Aside from these similarities, on a very general level, the socioeconomic and metabolic profiles of the two countries are quite different. A comparison of these two small countries, with their untypical metabolic profile, yields insights that help to advance the understanding of the emergence of metabolic profiles, the involved factors, and the interpretation of aggregate MFA indicators.

The development of the material flow patterns in TT and Iceland underlines the importance of resource endowment for the composition and size of material flows, from extraction to trade and domestic material consumption. Historically, both countries depended, to a high degree, on the extraction and export of renewable biomass, but shifted in the second half of the twentieth century toward the exploitation of mineral and fossil materials: Material flows in TT were initially influenced by the availability of fertile land, which was the basis for sugar production, and later, increasingly, by large deposits of petroleum and natural gas and the related material-intensive petrochemical and fertilizer industries. In the case of Iceland, the endowment with productive fishing grounds coined the metabolic profile until abundant hydropower and geothermal heat became the basis for the establishment of material-intensive metal industries. In this sense, both cases demonstrate how energy availability is a major factor driving material use.

But, the two cases also show that the endowment with exploitable resources is only one aspect. Of equal importance are infrastructures and specific institutional settings that facilitate resource extraction, processing, and export. They demonstrate how the availability of infrastructure for the exploitation of natural resources (e.g., pipelines and harbors to export oil and gas) or their lack (e.g., a submarine cable for electricity export) determine if and how a resource is exploited and used and in which way resource endowment influences the material flow profile. Next to these technical issues, institutional settings are of high significance: Resource use in TT was first driven by colonial land ownership patterns and then by TT's policy of industrialization by invitation paired with the United States’ import restrictions. In Iceland, the expansion of fishing grounds and the organization of fishing rights as well as bargains with multinationals concerning power supply, electricity prices, and tax conditions shaped the metabolic profile and economic development. Institutional arrangements determine if and how sustainable domestic resources are exploited and who profits from their exploitation. As a result of specific technological decisions and institutional settings, path dependencies can emerge with long-lasting effects on the physical economy.

In both TT and Iceland, a very small set of key materials dominate extraction and export. It is important to see that energy availability also has a large, indirect effect on material use: Both countries use their abundant energy resources (natural gas and hydropower) to exploit other resources or to run material-intensive processes. In the case of TT, natural gas is used in energy-intensive fertilizer and iron and steel production (Furlonge and Kaiser 2010); in Iceland, electricity fuels alumina and ferroalloy production (Valfells et al. 2004). These energy- and material-intensive processes not only drive patterns of DE and exports, but they also have a strong influence on imports and lead to a high DMC: Although a large part of the extracted and processed materials is exported, considerable waste flows remain in the country and contribute to high DMC, high energy consumption, and high CO_2_ emissions. To some degree abundance of particular resources is also related to above-average domestic final consumption of these resources, for example, the high per capita fish consumption or high household electricity consumption in Iceland.

The two case studies illustrate the complexity of the role of trade in sustainable development (Perez-Rincon 2006; Muñoz et al. 2011). Both countries exploited their natural resources for export production, which fueled their economic development, created wealth, and allowed them to import commodities not available domestically. This strategy was not always equally successful, but, in the long run, both countries improved the management of their resources and the benefits they were able to gain over time; though the exploitation of natural resources for exports caused a considerable loss of natural capital and was associated with ecological costs, it can be also argued that the establishment of the aluminum industry in Iceland, for example, is saving natural resources on the global scale because extraction and processing is more efficient and less CO_2_ intensive in Iceland than elsewhere.

The reliance on a few abundant material or energy resources results in high dependency on global markets. The two cases illustrate the vulnerability of small island economies, depending on the exploitation of a few key resources with respect to fluctuations in resource prices, access to markets, and overexploitation (Chertow et al. 2013). Whereas high resource prices boost the economy, falling prices or overexploitation and declining stocks can lead to severe economic problems: Fish prices and overfishing in Iceland (see Agnarsson and Arnason 1993), oil prices, and peak oil in TT (see Lorde et al. 2009) have repeatedly caused fierce economic crises in both countries. The high dependence on imports of food and other important consumer goods further increases this vulnerability.

Finally, our results highlight some of the strengths and weaknesses of aggregate MFA-derived indicators. On the one hand, it becomes clear that the exceptionally high per capita DMC in TT and Iceland is largely a result of waste flows related to resource-intensive export production (e.g., bauxite waste, high petroleum and gas consumption of petrochemical industries, and fish reduction); on the other hand, both countries import basically all their food, and the largest part of their consumer goods—and all the upstream resource requirements associated with the production of these commodities for final consumption—are not recorded in their DMC. Although these effects may balance in larger economies with more complex production and trade structures, for small open countries such as those studied here, this can lead exceptionally high or low DMC values (see also Schandl and West 2012). It is important to keep in mind that DMC measures apparent consumption, that is, it measures aggregate resource use of an economy, but it neither allows for differentiating between materials used to produce goods and services for domestic final consumption and for exports nor does it reflect resources used in the production of imported goods. To quantify consumption-related resource use, other instruments are needed and are being developed (Bruckner et al. 2012; Peters et al. 2011; Schaffartzik et al. 2014). But, from our analysis, it also became clear that DMC does provide important information about the material-use patterns resulting from the domestic production structures, structures that created a large part of the economic wealth in the two countries and provide labor and income for large fractions of the population. DMC thus informs those material flow patterns the countries are immediately responsible for, which they can influence directly by national politics and technology and which eventually become domestic waste flows (DMC as a measure of the domestic waste potential; see Weisz et al. [2006]).

## Footnotes

The MFA data for Iceland and Trinidad and Tobago are available for download at: www.uni-klu.ac.at/socec/inhalt/1088.htm.

According to current conventions, material flow accounts do not account for water and atmospheric gases. In cases where chemical reactions (e.g., combustion of energy carriers) or other processes (e.g., changes in the moisture content of materials) that involve atmospheric gases or water have an effect on the mass flow of materials, the corresponding mass flows of water and atmospheric gases are considered balancing items that close the mass balance. In general, balancing flows have a very minor impact on DMC and are therefore ignored in most material flow accounts. TT is an extreme case here because large amounts of exported nitrogen fertilizer are not counterbalanced by any domestic extraction. The involved flows are so large, that not including nitrogen as a balancing item in DE would result in negative values of DMC for the group of nonmetallic minerals.

There are no data available that allow for a straightforward allocation of material use to industrial or household consumption. For energy use and CO_2_ emissions, it is estimated that households are only responsible for 3%, transport for 6%, and industry including energy provision for 91% of energy use. Correspondingly, industry and energy provision are responsible for 84% of all CO_2_ emissions; only 1% of all CO_2_ emissions are a result of household energy consumption (Ministry of Energy and Energy Affairs [MEEA] 2011)

The loss of moisture (over 1 Mt/yr) must be considered a processing waste flow, which adds to the domestic material consumption according to the principles of MFA.

After Qatar, TT and Iceland are the countries with the highest energy use per capita (12.7 and 11.7 t oil equivalent per capita per year (IEA 2010).

## Supporting Information

Supporting Information S1 This supporting information provides the physical and monetary flows and terms of trade for Trinidad and Tobago and Iceland.
