# Supplementary material for: Resource Use in Small Island States: Material Flows in Iceland and Trinidad and Tobago, 1961–2008
Source: J Ind Ecol. 2014 Feb 12;18(2):294–305. doi: 10.1111/jiec.12100 (PMC4251509; doi:10.1111/jiec.12100)

SUPPORTING INFORMATION FOR:

Krausmann, F., R. Richter, and N. Eisenmenger. 2013. Resource use in small island states: Material flows in Iceland and Trinidad and Tobago, 1961-2008. *Journal of Industrial Ecology.*

Summary

This supporting information provides the physical and monetary flows and terms of trade for Trinidad & Tobago and Iceland.

Figure S1: Physical and monetary trade flows and terms of trade in Trinidad & Tobago (A) and Iceland (B). Sources: Physical trade flows in million tonnes (mio t): see text; monetary trade flows in billion Dollars (bio $): The World Bank Group 2012 in constant US$ of the year 2000; terms of trade: own calculation based on physical and monetary trade flows.

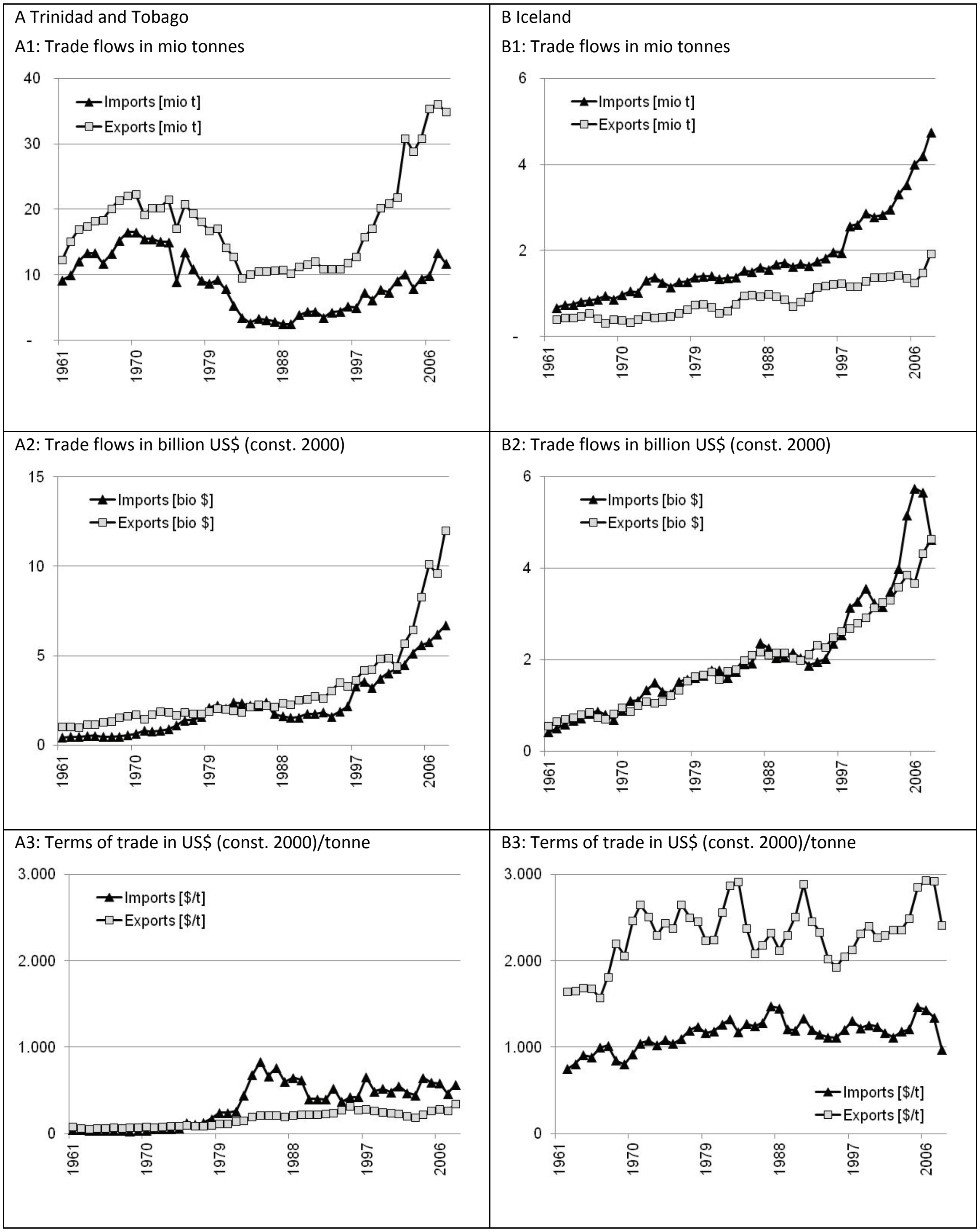

Supplement: Supplementary file 1 — Supporting info item [file 44498_2014_1802013_MOESM1_ESM.pdf]
